# Seasonal Effect on Disease Onset and Presentation in Anti-MDA5 Positive Dermatomyositis

**DOI:** 10.3389/fmed.2022.837024

**Published:** 2022-02-04

**Authors:** Ho So, Jacqueline So, Tommy Tsz-On Lam, Victor Tak-Lung Wong, Roy Ho, Wai Ling Li, Chak Sing Lau, Lai-Shan Tam

**Affiliations:** ^1^Department of Medicine and Therapeutics, The Chinese University of Hong Kong, Shatin, Hong Kong SAR, China; ^2^Department of Medicine and Therapeutics, Prince of Wales Hospital, Shatin, Hong Kong SAR, China; ^3^Department of Medicine and Geriatrics, Kwong Wah Hospital, Kowloon, Hong Kong SAR, China; ^4^Department of Medicine, Queen Elizabeth Hospital, Kowloon, Hong Kong SAR, China; ^5^Department of Medicine, Queen Mary Hospital, Hong Kong, Hong Kong SAR, China; ^6^Department of Medicine, The University of Hong Kong, Pokfulam, Hong Kong SAR, China

**Keywords:** anti-MDA5, dermatomyositis, idiopathic inflammatory myopathy, myositis specific antibody, rapidly progressive interstitial lung disease (RP-ILD)

## Abstract

**Objective:**

To investigate the seasonal variation of disease onset and presentation in an ethno-geographically homogeneous cohort of patients with anti-MDA5 positive dermatomyositis (DM).

**Methods:**

This was a multi-centered, retrospective cohort study. Adult Chinese anti-MDA5 positive DM patients were identified from the Hong Kong Myositis Registry and the Clinical Data Analysis and Reporting System from 2015 to 2020. Equal number of IIM patients without anti-MDA5 antibody were selected as controls. Line blot immunoassay was used to detect the autoantibodies. The onset of disease, presenting clinical features and subsequent complications were analyzed for any seasonality.

**Results:**

A total of 110 patients with anti-MDA5 positive DM were studied. The mean age at diagnosis was 53.0 ± 12.3 years and the mean follow-up duration was 20.6 ± 23.1 months. Two third of the patients (66%) had the clinically amyopathic phenotype. Most patients (86%) had interstitial lung disease (ILD) and 42% developed rapidly progressive ILD (RP-ILD). The mortality was 40% and the commonest cause was RP-ILD. Chi-square test showed significantly less patients had symptom onset in July to September. However, no particular seasonal pattern was observed in the anti-MDA5 negative IIM controls. RP-ILD occurred more frequently in patients with disease onset in October to December. Anti-MDA5 positive DM patients with disease onset in warmer months (April to September) were more likely to have clinical muscle involvement.

**Conclusion:**

Apparent seasonal patterns were noted in our ethno-geographically identical anti-MDA5 positive DM patients, but not in IIM patients in general. Certain environmental factors, particularly infection, might be implicated.

## Introduction

The etiology of idiopathic inflammatory myopathies (IIMs) is largely unknown. It is generally believed that a variety of environmental factors, acting upon different genetic backgrounds (HLA or cytokine polymorphisms), result in distinct immune responses and clinical syndromes in IIMs (1). Identifiable seasonality could shed light on the etiology and enhance our understanding of the disease pathogenesis. It could also increase our awareness of the disease and its complications during the peak seasons. However, seasonal patterns of disease onset and severity in IIMs as a whole are conflicting.

In recent years, over 10 myositis-specific antibodies (MSAs) have been identified. They are able to divide IIM patients into homogenous subgroups and inform prognosis (2). Anti-melanoma differentiation-associated protein 5 (MDA5) positive dermatomyositis (DM) attracts special clinical attention due to its strong association with rapidly progressive interstitial lung disease (RP-ILD) which carries a high mortality (3). Patients with the disease also present with unique clinical features such as refractory vasculitic rash and amyopathy (4, 5). There seems to be a geographical or ethnic variation in the prevalence and phenotype of anti-MDA5 positive DM (6–8). In this study, we aimed to investigate the seasonal pattern of disease onset and presentation in an ethno-geographically identical cohort of patients with anti-MDA5 positive DM.

## Methods

### Study Design and Patients

This was a multi-centered, retrospective cohort study conducted in 10 regional hospitals in Hong Kong. The primary objective of the study was to investigate the seasonal variations of onset of anti-MDA5 positive DM in Chinese patients. The secondary objective was to examine the seasonal patterns of various clinical manifestations of the disease.

Consecutive IIM patients diagnosed between January 2015 to December 2020 were identified from the Hong Kong Myositis Registry (MyoHK) and the Clinical Data Analysis and Reporting System (CDARS). MyoHK is a territory-wide registry that was set up in 2019 aiming to systemically collect clinical information of IIM patients in Hong Kong. CDARS is an electronic system created by the Hong Kong Hospital Authority since 1991, mainly for audit and research purpose. The system has been extensively used in large-scale epidemiological studies (9–11). Clinical records of patients identified via MyoHK and CDARS were reviewed by the investigators individually via electronic health record (EHR) to screen for eligibility for recruitment into the study. Inclusion criteria included: (1) adult onset disease (≥18 years), (2) Chinese ethnicity, (3) residing in Hong Kong, (4) diagnosis of DM based on Bohan and Peter's criteria or EULAR/ACR 2017 classification criteria, (5) diagnosis of CADM defined as typical cutaneous features of DM confirmed by rheumatologist or dermatologist but minimal or no clinical features of myositis (hypomyopathic and amyopathic) according to the Sontheimer's criteria, and (6) a positive anti-MDA5 antibody result (12–14). Exclusion criteria included: (1) myositis secondary to trauma, seizure, alcohol or drug abuse or myopathic drugs, such as statin and fibrates, and (2) patients with other concomitant connective tissue diseases. Equal number of IIM patients diagnosed in the similar period without anti-MDA5 antibody were randomly selected as controls. A commercial line blot immunoassay kit (Euroline Autoimmune Inflammatory Myopathies 15 Ag, Euroimmun, Lubeck, Germany) was used to detect the MSAs. The study was approved by the local research ethics committees and patient consents were waived due to the retrospective nature of the study.

### Disease Variables

All participating hospitals used the same EHR system for clinical information documentation. Data of the recruited patients were reviewed and collected via the EHR by the investigators from the date of IIM diagnosis until either death or the end of study (31st December, 2020). These included patient demographics, onset of disease, presenting clinical characteristics and subsequent complications. ILD was defined by typical radiological features of ILD on computer tomography (15). RP-ILD was defined as worsening of radiologic interstitial change, progressive dyspnoea and hypoxemia within 1 month of onset of respiratory symptoms (16, 17). Infections were all microbiologically proven.

### Statistical Analysis

Descriptive statistics were presented as frequencies, means with standard deviation or medians with ranges as appropriate. Seasonal patterns were initially evaluated using circular statistics with Rayleigh's test of the average vector magnitude. The effect of seasonality for onset of disease was then analyzed by Chi-square test with the calendar months of a year categorized into four seasons: spring (April to June), summer (July to September), autumn (October to December) and winter (January to March) roughly, according to the climatological information of Hong Kong (Supplementary Figure 1). Comparisons of various clinical manifestations in different seasons were done using Chi-square test or Fisher exact test for categorical variables, independent samples *t*-test or Mann-Whitney U test for continuous variables. Results were considered statistically significant if the *p*-value was < 0.05. Statistical analyses were performed by using the Statistics Package for Social Sciences (SPSS inc., Chicago, version 24.0) and R3.3.1 software.

## Results

All together 110 patients with anti-MDA5 positive DM were recruited (Table 1). The mean age of the anti-MDA5 positive patients at diagnosis was 53.0 ± 12.3 years and the mean follow-up duration was 20.6 ± 23.1 months. There was a slight female predominance (56%). Two third of the patients had the CADM phenotype (66%). Common presenting symptoms included arthritis (64%), fever (49%), weight loss (38%), vasculitic rash (37%), and infection (23%). The list of infections at presentation is shown in Table 2. The great majority of the patients (86%) had ILD and 42% developed RP-ILD. Most of the patients with RP-ILD (40/46, 87%) developed this condition within 3 months of the diagnosis of DM. Other complications included refractory rash (62%), hoarseness of voice (15%), dysphagia (14%), pneumothorax (14%), pneumomediastinum (9%), leucoplakia (9%), cancer (5%) and cardiomyopathy (4%). The overall mortality of patients with anti-MDA5 related DM was 40% (44/110) with the commonest cause being RP-ILD.

**Table 1:** The demographics, clinical characteristics, and complications of 110 anti-MDA5 positive DM patients.

	***N* = 110**
Female, no. (%)	61 (56)
Age of onset, years, mean (±SD)	53.0 (12.3)
Duration of follow-up, months, mean (±SD)	20.6 (23.1)
Smoker, no. (%)	9 (8)
Subtypes
- DM, no. (%)	37 (34)
- CADM, no. (%)	73 (66)
- PM, no (%)	0 (0)
ILD, no. (%)	95 (86)
RP-ILD, no. (%)	46 (42)
Arthritis, no. (%)	70 (64)
Fever at presentation, no. (%)	54 (49)
Weight loss, no. (%)	42 (38)
Vasculitic rash, no. (%)	41 (37)
Hoarseness of voice, no. (%)	16 (15)
Leukoplakia, no. (%)	10 (9)
Infection at presentation, no. (%)	26 (24)
Dysphagia, no. (%)	15 (14)
Pneumothorax, no. (%)	15 (14)
Pneumomediastinum, no. (%)	10 (9)
Malignancy, no. (%)	5 (5)
Cardiac involvement, no. (%)	4 (4)
Mortality, no. (%)	44 (40)

*MDA5, melanoma differentiation-associated protein 5; DM, dermatomyositis; CADM, clinically amyopathic dermatomyositis; PM, polymyositis; ILD, interstitial lung disease; RP-ILD, rapidly progressive interstitial lung disease*.

**Table 2:** Documented infections at presentation of 110 anti-MDA5 positive DM patients.

	**N (%)**
Sites
- Respiratory tract	16 (15)
- Blood	4 (4)
- Skin	3 (3)
- Urinary tract	1 (1)
Organisms:
- *Staphylococcus Aureus*	4 (4)
- *Pseudomonas Aeruginosa*	3 (3)
- *Haemophilus Influenzae*	2 (2)
- *Klebsiella pneumoniae*	2 (2)
- *Salmonella species*	2 (2)
- *Citrobacter*	1 (1)
- *Enterobacter*	1 (1)
- *Escherichia coli*	1 (1)
- *Morexella catarrhalis*	1 (1)
- *Morganella morganii*	1 (1)
- *Stenotrophomonas maltophilia*	1 (1)
- *Serratia marcescens*	1 (1)
- *Rickettsia*	1 (1)
- *Mycobacterium Abscessus*	1 (1)
- *Pneumocystis jirovecii*	1 (1)
- *Candida species*	1 (1)

**Figure 1:**
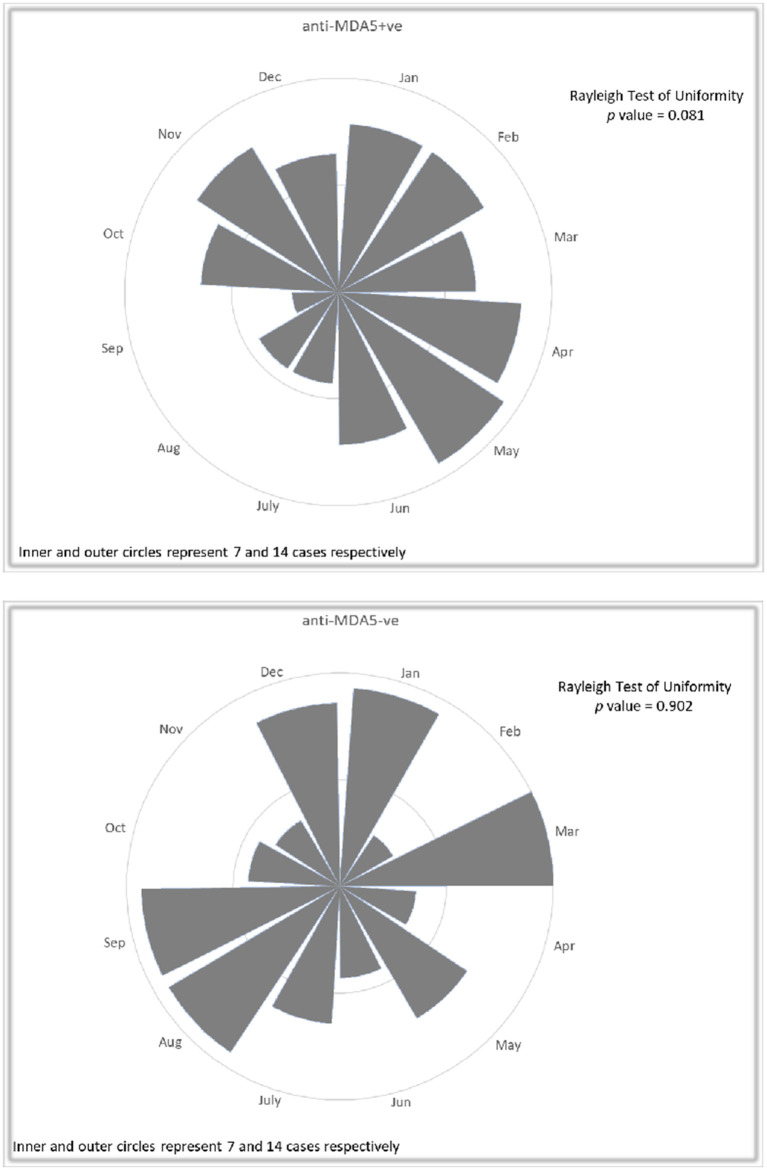
Rose diagrams with Rayleigh's test in 110 patients with anti-MDA5 positive DM and 110 anti-MDA5 negative IIM controls with regard to disease onset. A pattern of less frequent anti-MDA5 positive DM patients in July to September was noted, while no particular trend was observed in anti-MDA5 negative IIM patients.

Rayleigh's test and rose diagram revealed a pattern that less anti-MDA5 positive DM patients had disease onset in summer months (July to September), whereas no particular trend was observed in anti-MDA5 negative IIM patients (Figure 1). Chi-square test confirmed that significantly less anti-MDA5 positive patients (observed number 15, excepted number 27.5, *p* = 0.041) developed initial symptom in July to September. No seasonality difference in disease onset was observed in the anti-MDA5 negative patients (*p* = 0.19), among them 19% had anti-TIF1, 18% had anti-synthetase, 9% had anti-SRP, 8% had anti-Mi2, 4% had anti-SAE, 3% had NXP2, 2% had anti-HMGCR antibodies and 40% were MSA negative. When we compared disease onset in anti-MDA5 positive patients with RP-ILD and patients without, it was found that while both groups had a dip in summer season from July to September (with RP-ILD: observed number 5, expected number 11.5, *p* = 0.032; without RP-ILD: observed number 10, excepted number 16, *p* = 0.028), a peak was noted from October to December only in the RP-ILD group (observed number 19).

RP-ILD occurred significantly more frequently in patients with disease onset in the autumn (October to December) months (spring: 34%, summer: 33.3%, autumn 66%, winter: 32%; *p* = 0.028). Further analysis showed that infections were significantly associated with the development of RP-ILD (*p* = 0.02). On the other hand, anti-MDA5 positive DM patients with disease onset in colder months (October to March) were less likely to have clinically significant weakness (spring: 49%, summer: 47%, autumn: 31%, winter: 13%, *p* = 0.013) and hoarseness (spring: 20%, summer: 40%, autumn: 0%, winter: 10%, *p* = 0.003). No other significant seasonality was observed in other clinical presentations or complications.

## Discussion

Seasonal variations have been observed in many rheumatic diseases regarding incidence, relapse and severity (18, 19). However, seasonal patterns of disease onset in IIM patients as a whole or in the traditional polymyositis (PM) or DM populations are conflicting. In a very early report on seasonal distribution in the onset of the disease of 51 cases of PM/DM, a concentration of cases was found for the months of March, April and May (20). In an Australian study of 53 patients with treated IIMs, relapses involving, both muscular and cutaneous, occurred more often during the summer and spring in DM, but no significant seasonal trends were found in PM patients (21). However, in a larger study of 200 patients with IIMs, no recognizable seasonal patterns could be found in the conventional categories of PM and DM (22). Interestingly, it was shown that the month of weakness onset was not random in patients with anti-Jo-1 autoantibody (average month April) and in those with anti-SRP autoantibody (average month November). In a subsequent study of 503 patients, again no significant seasonal patterns of disease onset in IIM patients as a whole or in the total PM or DM populations were found (23). Significant seasonal associations were present, however, in the serologically defined groups with patients having anti-synthetase antibodies peaked in March to April and patients not having any autoantibodies peaked in June to July. These findings suggest that searches for seasonal patterns in the onset of IIM characterized by MSAs may provide more useful clues to etiology of the disease.

We conducted a multi-centered study of a homogeneous disease group, based on ethnicity, residential area and serology, aiming to investigate the seasonality of IIMs. It was found that anti-MDA5 DM patients presented significantly less frequently in July to September which corresponded to the summer season in Hong Kong, whereas no clear seasonal pattern was observed in anti-MDA5 negative IIM controls. Patients who developed RP-ILD, which was shown to be associated with infection, often had disease onset in the colder months (October to December). This seasonal pattern appears similar to that of the respiratory virus infections (24, 25). A viral trigger for IIM has attracted much attention, because of considerable circumstantial evidence: the frequent reports by patients that their symptoms were preceded by an acute “viral” illness, elevated titres of antibodies to certain viruses in patients with myositis, presence of viral RNA in muscle biopsy specimens (26–29). A Swedish case–control study also reported preceding gastrointestinal and respiratory tract infection as a risk factor for IIM (30). In fact, being an important member of the cytoplasmic RIG-I like receptor family that functions in innate immunity, MDA5 recognizes intracellular viral nucleic acids and triggers type I interferon production that leads to suppression of viral replication. Thus, it has been postulated that anti-MDA5 related DM is triggered in genetically predisposed individuals following exposure to viral infections, which elicits loss of self-tolerance and antibodies production, which in turn could create a self-perpetuating autoimmune cycle with lungs being the primarily triggering and affected organ (31). On the other hand, significant muscle involvement and hoarseness, which is presumably related to pharyngeal muscle weakness, were more frequently noted in anti-MDA5 positive DM patients with disease onset in warmer months. Whether this could represent a difference in etiology or triggering factor in patients with muscle and pulmonary involvement warrants further investigations.

A single-center study in Japan reported that CADM patients were less prevalent in urban areas and the majority of anti-MDA5-positive patients resided in rural areas around a large river (32). The same study also showed that the incidence of anti-MDA5 antibodies in less populated areas, but not in areas with populations over 130 × 10^3^, was the highest in autumn. In a subsequent large multi-centered Japanese study in patients with IIM related ILD, it was found that anti-MDA5 positive patients had disease onset predominantly from October to March, whereas no seasonality was noted in patients with anti-synthetase antibody or tested negative for the above two antibodies (33). A preferential geographical distribution of anti-MDA5-assoicated ILD near freshwater was again noted. In a research letter, a summer dip in the incidence was also reported in a French cohort of DM patients with anti-MDA5 antibody (34). All these support the postulation that environmental factors, particularly infectious agents, could contribute to the production of autoantibodies against MDA5. However, a study using data from a North American registry reported no seasonal clustering of the month of diagnosis in 35 juvenile IIM patients with anti-MDA5 antibody (35). The discrepancy could be related to the juvenile disease subgroup, ethno-geographical difference or relative small sample size. Interestingly, Hosono et al. identified a novel autoantibody against splicing factor proline/glutamine-rich protein (SFPQ), which is known to play a role in innate immune responses, in patients with anti-MDA5 antibody-positive DM (36). These patients had disease diagnosis showing seasonal patterns according to the timing of anti-SFPQ antibody appearance suggesting that autoantibody production may also have some association with environmental factors. In this cohort of patient with both antiMDA5 and anti-SFPQ antibodies (*N* = 27), no patients had disease onset in summer season (June and July). In our study, residential clustering was not formally studied, as Hong Kong is a relatively geographically restricted city. The number of cases contributed by each regional center was proportionate to the population it served [data not shown].

There are several limitations in this study. First, despite the increasing popularity, the line blot immunoassay has not been fully validated. Second, due to the retrospective nature of the study, recall bias and incomplete data collection were inevitable. Importantly, the exact onset of symptoms related to the disease could be poorly defined which might significantly affect the seasonal patterns identified. Third, respiratory viral infection testing was not routinely performed limiting the examination of the potential inciting agents. Lastly, other potential mechanisms for the seasonal variation in the disease pattern including climatic differences and UV light exposure were not examined (37).

To conclude, apparent seasonal patterns were noticed in our ethno-geographically identical anti-MDA5 related DM patients, but not in anti-MDA5 negative IIM controls. We found a concentration of cases particularly those with significant lung involvement in colder months. Further studies to identify the possible environmental etiologic factors are encouraged.

## Data Availability Statement

The raw data supporting the conclusions of this article will be made available by the authors, without undue reservation.

## Ethics Statement

The studies involving human participants were reviewed and approved by The Chinese University of Hong Kong—New Territories East Cluster Clinical Research Ethics Committee, No. 2020-0743; New Territories West Cluster Research Ethics Committee, No. 20132; Hong Kong East Cluster Clinical Research Ethics Committee, No. 2021-0010; Hong Kong West Cluster Research Ethics Committee, No. 2021-0037; Research Ethics Committee (Kowloon Central/Kowloon East), No. 2021-0022; Kowloon West Cluster Research Ethics Committee, No. 2020-0194. Written informed consent for participation was not required for this study in accordance with the national legislation and the institutional requirements.

## Author Contributions

HS, CSL, and L-ST: study design. HS, JS, TT-OL, VT-LW, RH, and WLL: data collection. HS and JS: data analysis. JS, HS, CSL, and L-ST: drafting of manuscript. All authors critically revised the manuscript for important intellectual content.

## Conflict of Interest

The authors declare that the research was conducted in the absence of any commercial or financial relationships that could be construed as a potential conflict of interest.

## Publisher's Note

All claims expressed in this article are solely those of the authors and do not necessarily represent those of their affiliated organizations, or those of the publisher, the editors and the reviewers. Any product that may be evaluated in this article, or claim that may be made by its manufacturer, is not guaranteed or endorsed by the publisher.
