# Supplementary material for: Seasonal Effect on Disease Onset and Presentation in Anti-MDA5 Positive Dermatomyositis
Source: Front Med (Lausanne). 2022 Feb 4;9:837024. doi: 10.3389/fmed.2022.837024 (PMC8854504; doi:10.3389/fmed.2022.837024)
Supplement: Supplementary file 1 [file Data_Sheet_1.docx]

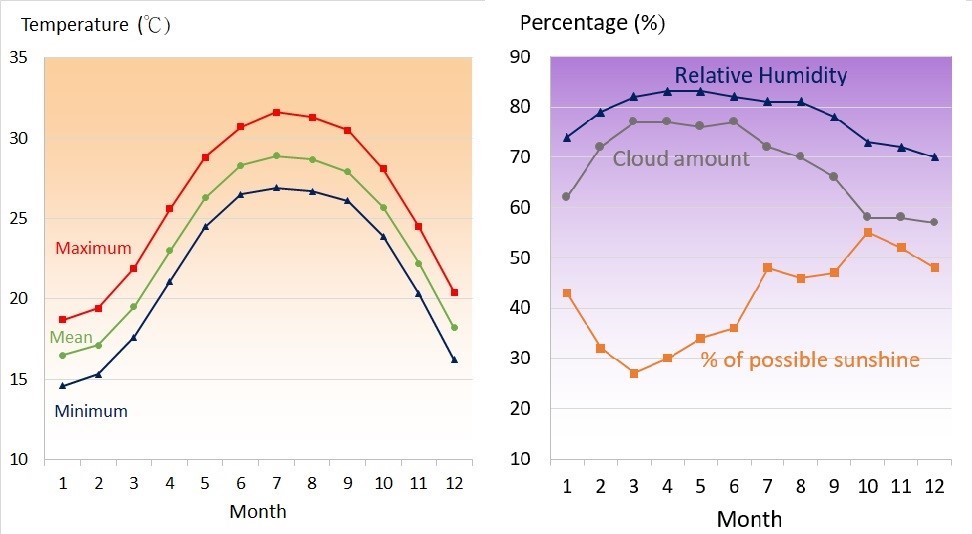


**Supplementary figure 1.** Monthly means of daily maximum, mean and minimum temperature (left), relative humidity, cloud amount and percentage of possible sunshine (right) in Hong Kong between 1991-2020.
